# Provider Perspectives on Implementing an Enhanced Digital Screening for Adolescent Depression and Suicidality: Qualitative Study

**DOI:** 10.2196/67624

**Published:** 2025-04-10

**Authors:** Morgan A Coren, Oliver Lindhiem, Abby R Angus, Emma K Toevs, Ana Radovic

**Affiliations:** 1 School of Medicine, University of Pittsburgh, 100 N Bellefield, Office 537, Pittsburgh, PA, 15213, United States, 1 412-2465909; 2 University of Pittsburgh Medical Center, Western Psychiatric Hospital, Pittsburgh, PA, 15213, United States; 3 Adolescent and Young Adult Medicine, University of Pittsburgh Medical Center, Children's Hospital of Pittsburgh, Pittsburgh, PA, United States

**Keywords:** depression, suicidality, adolescent mental health screening, primary care, digital tools

## Abstract

**Background:**

With a growing adolescent mental health crisis, pediatric societies are increasingly recommending that primary care providers (PCPs) engage in mental health screening. While symptom-level screens identify symptoms, novel technology interventions can assist PCPs with providing additional point-of-care guidance to increase uptake for behavioral health services.

**Objective:**

In this study, we sought community PCP feedback on a web-based, digitally enhanced mental health screening tool for adolescents in primary care previously only evaluated in research studies to inform implementation in community settings.

**Methods:**

A total of 10 adolescent providers were recruited to trial the new screening tool and participate in structured interviews based on the Consolidated Framework for Implementation Research domains. Interviews were audio recorded, transcribed, and coded according to a prespecified codebook using a template analysis approach.

**Results:**

Providers identified improving mental health screening and treatment in pediatric primary care as a priority and agreed that a web-based digitally enhanced screening tool could help facilitate identification of and management of adolescent depression. Salient barriers identified were lack of electronic health record integration, time to administer screening, implications on clinic workflow, accessibility, and lack of transparency within health care organizations about the process of approving new technologies for clinical use. Providers made multiple suggestions to enhance implementation in community settings, such as incorporating customization options.

**Conclusions:**

Technology interventions can help address the need for improved behavioral health support in primary care settings. However, numerous barriers exist, complicating implementation of new technologies in real-world settings.

## Introduction

Adolescent depression and suicidality are part of a growing public health crisis that has been further exacerbated by the COVID-19 pandemic [1]. In 2022, 19.5% of US adolescents aged 12‐17 years experienced a major depressive episode and 13.4% reported having seriously considered suicide [2]. Pediatric societies recommend that pediatric primary care providers (PCPs) screen adolescents for both depression and suicidality [3-6]. Although screening adolescents for depression is recommended by the US Preventive Services Taskforce [7], there is no evidence to support that screening by itself improves health outcomes [7]. PCPs recognize the need for additional interventions to increase uptake of services and provide clinical benefit, but requirements for increased mental health staff and support obfuscate their implementation.

Technology interventions can aid providers to implement screening recommendations and augment initiatives to improve behavioral health support in primary care. In recent years, technology-based screeners have been developed for detecting conditions in addition to depression such as posttraumatic stress disorder (PTSD) [8], substance use [9], and interpersonal violence [10] in primary care settings, as well as to screen patients for social needs [11]. Such interventions have proven to be effective, accurate, well-received by patients, and helpful in streamlining clinic workflow and history-taking.

**Figure 1.**
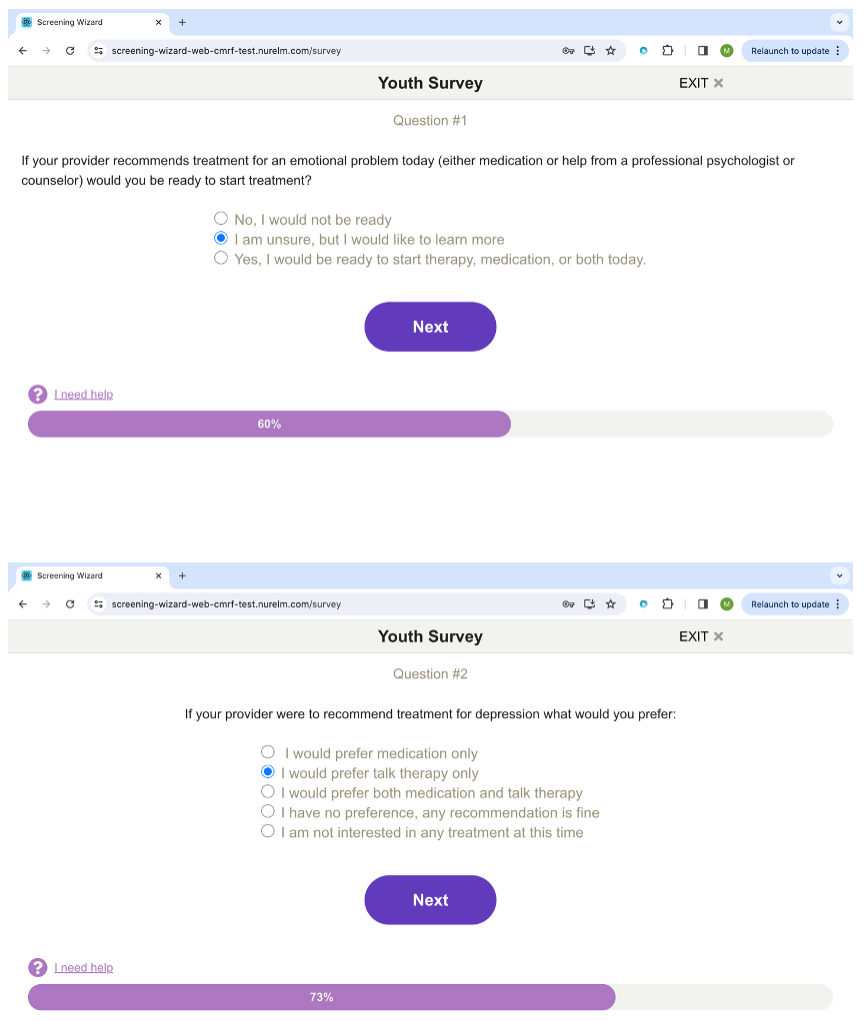
Sample images of the Screening Wizard tool, as it appears to patients.

Screening Wizard (SW; see Figure 1 for a screenshot) is a web-based digitally enhanced screening tool for eliciting symptoms of depression and suicidality in adolescents. In addition to surveying adolescents and their caregivers through separate unique weblinks with validated screening measures for comorbidities of anxiety and mania, the screening tool assesses additional considerations, such as treatment readiness, motivation, treatment preferences, and barriers. The surveys take approximately 5‐7 minutes to complete. Once completed, a report succinctly synthesizes the information to provide PCPs with comparisons between adolescents and their caregiver responses that may guide management decisions and referrals. The initial qualitative study informing the design of SW [12], evaluation of iterative feedback from stakeholders, and pilot usability trial of the screening tool in a general sample of adolescents [13] are described in previously published papers.

These previous studies and provider feedback informed several modifications to the enhanced screening tool so that it could be used clinically outside of a research context. The clinical use version was updated with functionality for a clinic-specific Health Insurance Portability and Accountability Act (HIPAA)–secure log-in to the SW system to separate its administration and data collection from the SW research environment. This allowed clinical staff to assign adolescent patients and their caregiver unique weblinks to the enhanced screen and schedule when they would receive it through text message directly to their mobile phone; or alternatively print a list of QR code weblinks to distribute to individual patients. After completion, staff would be able to review results on a private clinician dashboard. In response to safety and liability concerns related to remote suicide screening, surveys were scheduled to expire at the end of the same clinic day to allow time for clinic staff to review all completed surveys. Planned changes also included condensing the number of items by presenting additional screening items only for participants with positive initial depression screens. Integration of the screening tool into the electronic health record (EHR) had been identified as a high priority initiative at this stage, though this required clinical system-wide permission, which was not accessible to the research team.

As the enhanced screening tool was initially developed within an academic setting with access to embedded behavioral health staff and research support, this study was intended to pilot the screening tool in more typical and less-resourced community health care settings where adolescents seek primary care or other services (eg, reproductive care) to elucidate barriers and facilitators to implementation from providers who are the intended users of the tool. This tool was offered to multiple clinical sites to pilot and provide feedback on the piloting process. Although health care providers at these sites were interested in using the intervention, many clinics (especially those within larger health care systems) had extensive IT audit and administrative approval processes to trial new technology tools, which presented substantial barriers to piloting. Only 3 out of 10 clinics were able to pilot the tool very briefly. It became apparent that barriers to implementation would differ in a real-world versus research setting and additional elicitation from providers about these barriers and facilitators would be needed to enable scalability. Preimplementation studies are most helpful when they allow participants to try out the tool [12,14]. However, due to the barriers to piloting, we developed an additional prototype of the tool for interested providers to test informally, using fabricated patient data.

In this study, we conducted structured interviews with primary care providers based on domains from the Consolidated Framework for Implementation Research [15,16], a widely used framework for systematically identifying barriers and facilitators to implementation of new interventions. The information learned will be used to inform future implementation efforts and make subsequent iterations of the enhanced screening tool better suited for use in a variety of practice settings. This paper describes our qualitative process and the feedback we received.

## Methods

### Participants

A total of 10 providers from 10 different clinical sites across the United States were recruited through purposive sampling to participate in structured interviews. Providers were included if they provided either primary care or specialty adolescent medicine care (eg, for reproductive, eating disorder, and gender concerns) to the adolescent age group. They were excluded if their clinic had participated in previous studies with the screening tool. Providers were recruited from primary care clinics within the University of Pittsburgh Medical Center network who had not participated in the previous study, and from a national organization adolescent medicine listserv where providers had previously expressed interest in trialing the clinical tool after Dr Radovic had replied to a member’s question about existence of available computerized clinical tools.

### Ethical Considerations

Participants provided verbal consent to participate in interviews and were compensated with a US $50 gift card. Participants and interview content were deidentified. This study was approved by the University of Pittsburgh institutional review board (STUDY21090136).

### Procedure

Interested providers who were able to obtain organizational clearance were invited to trial the digitally enhanced mental health screening tool with actual patients, or if not, to informally explore a prototype of the tool on their own before the interview. All participants subsequently participated in a structured interview. Interview sessions were conducted by the first author through HIPAA-compliant Zoom (Zoom Communications) and comprised of a brief live demonstration of the screening tool followed by a 45-minute structured interview using a guide based on the Consolidated Framework for Implementation Research (CFIR) constructs [16] and informed by pilot work [13]. Table 1 shows the examples of interview questions.. The complete interview guide is available in Multimedia Appendix 1.

**Table 1.** Sample interview questions about a digitally enhanced mental health screening tool and their correspondence to the consolidated framework for implementation research.

CFIR^a^ domain	Construct	Interview question
Inner setting	Compatibility	How well does the enhanced screening tool fit with existing screening practices in your clinic?
Process	Engaging	Who are the key influential individuals to get on board with the enhanced screening tool?
Intervention characteristics	Adaptability	What changes or alterations do you think would be needed to facilitate implementation and utility of the screening tool in your clinic?

a CFIR: Consolidated Framework for Implementation Research.

### Coding and Qualitative Analysis

Interviews were audio-recorded, deidentified, and transcribed using Rev Human Transcription Services [17], removing identifiers and filler words (eg, “yeah”). Meeting notes were taken by the interviewer in real time for triangulation with eventual formal qualitative coding. Transcripts were coded by the first author following a template analysis approach [18] with NVivo software (version 14; Lumivero), using a prespecified codebook. The codebook was created according to a hybrid approach, using a deductive approach to generate high-level codes based on the CFIR Codebook Template [15], and an inductive approach to incorporate themes that arose from the data [19]. Subcodes were chosen based on topics recurring as key points across multiple interviews, and codes that did not fall within the CFIR framework were included on a case-by-case basis as relevant to future implementation of the screening tool. A research assistant coded data in parallel, and the 2 coders discussed and resolved discrepancies, with the availability of the senior author in case a resolution could not be found. The 2 coders had differences in organization of coding (for example, coding small sentence fragments versus coding complete blocks of text), but did not disagree on any content-level coding. All of these organizational discrepancies were resolved between the two coders and did not require escalation to the senior author. After consensus was reached between the two coders, the first author summarized and synthesized interview findings to present thematically. Participants were offered the opportunity to review findings presented in this manuscript and provide feedback for triangulation purposes. Participants did not suggest any corrections to the study’s findings.

## Results

Providers’ feedback regarding the digitally enhanced mental health screening tool and its theoretical implementation in their respective practice settings focused on four major themes: (1) current screening practices, (2) perceived benefits of the tool, (3) barriers to implementation, and (4) structural or organizational considerations. We reached saturation around these major themes.

### Description of Providers and Current Screening Practices

Of the providers contacted, 22 were invited by email to participate in interviews, 12 were interested in participating, and 10 ultimately completed interviews. All 10 providers viewed a prototype of the screening tool, and 3 of the providers attempted to pilot the tool in their respective clinics with patients but were unable to gain the required approval to do so. Of the 10 participants, 9 were physicians and 1 was a nurse practitioner. In addition, 8 of the providers specifically practiced adolescent medicine, while 2 providers worked in general pediatrics. In total, 7 providers practiced in hospital-based clinics associated with academic institutions, in a combination of primary care and adolescent specialty care settings. Furthermore, 1 provider worked in a college health clinic, and 2 providers practiced in community practices, with 1 working in a Federally Qualified Health Center.

In total, 6 of the 10 providers described using paper screeners for mental health, which clinic staff manually enter into the EHR, either by filling in a template with the patients’ responses or scanning the screening result into the EHR as a media file. In addition, 2 providers used an electronic screening format, whereby patients answer questions electronically and these answers are automatically integrated into the EHR. Furthermore, 1 provider used a combination of paper and electronic screening modalities, and another reported no formalized mental health screening process.

### Perceived Benefits of Digitally Enhanced Mental Health Screening Tool

Providers universally reported an uptick in adolescent depression and anxiety in recent years, particularly during the COVID-19 pandemic. Most providers supported mental health screening and treatment as high priority initiatives, with several reporting recent expansion of their practice’s mental health services (eg, hiring new embedded mental health providers and partnering with telepsychiatry services). All providers perceived the digitally enhanced mental health screening tool would offer improvements on their current practices due to it being administered through mobile devices before appointments and eliciting more detailed information than currently used screeners. Providers generally had positive opinions about any new intervention that could increase efficiency of history-taking, allowing a larger proportion of appointment time to be devoted to management discussions. Some providers recognized potential benefits of the tool in assisting with diagnostic clarity due to its efficiency in eliciting additional information without relying on a longer appointment. Previewing the tool and the included measures inspired one provider to inquire about mania in an adolescent patient with depression:

I thought a lot about the mania [screener], because it’s definitely not something that I traditionally screen for, unless I’m starting somebody on an SSRI…however, one of [my] patients screened positive for mania…I would not necessarily have thought to ask her those questions at that point, because we hadn’t even really started talking about medication yet. So, it definitely did prompt me to [ask further questions. The patient then revealed a strong family history of bipolar disorder], and I was like, “Oh, okay. This is starting to make more sense.” I think she actually does have bipolar type one.

The tools’ summarized results accompanied with additional symptom severity interpretation were recognized as efficiencies providers could use to identify mental health concerns at a glance and access direct links to current clinical care guidelines (ie, from the American Academy of Child and Adolescent Psychiatry and the American Academy of Pediatrics). One provider thought the tool would increase their comfort addressing mental health concerns in primary care, reflecting a lack of prior mental health training despite the need to address it regularly:

I wasn’t trained on how to use SSRIs or even stimulant medication really, even though I knew I was going to primary care and I tried to get that training and now that’s what I do a few times a day, which is kind of crazy.

Providers proposed that if the tool is introduced as a standardized screening that all adolescents and caregivers within the practice are asked to complete, it could normalize mental health discussions while simultaneously providing psychoeducation. Adopting the screening tool into their clinical practice was mentioned by a provider as a way of “letting people know that this [answering questions about their mental health] is something okay to talk about even when they’re not [currently symptomatic],” providing “a sense of safety and comfort for the patient.” Another provider, who regularly precepts pediatric residents, appreciated that the tool automatically engages parents in conversation about mental health, stating that this is something trainees struggle to facilitate on their own. Another provider relayed the tool could help dismantle mental health stigma, as many parents show heightened concern navigating the lasting impacts of the COVID-19 pandemic on youth:

There’s still a lot of stigma associated with mental health, but I think more and more people are getting more open, and I think a lot of parents will be very open to it right now, just given the current environment we’re in, understanding that a lot of young people and teens, everybody needs help once in a while, and it’s not something to be stigmatized.

Providers thought the enhanced screening tool could be used in various patient visit types besides annual well child visits, including as a diagnostic aid for patients presenting with mental health chief complaints, given the tool’s inclusion of several validated measures and specific questions regarding the patient’s treatment preferences and perceived barriers to care; and as a college health clinic administered prescreening for transitional age youth upon entering college to facilitate parental involvement in students’ transition to adult care.

### Barriers to Implementation

The most frequently mentioned theme throughout provider interviews was that implementing a new digital screening, which is not embedded in the EHR would be exceedingly challenging, requiring clinical staff to take on burdensome tasks including scheduling and sending out survey links, monitoring responses, printing results for providers, and uploading results into the EHR. This concern was in the setting of clinics already struggling with staffing shortages, and the perspective that—while highly important—mental health screening is only one facet of well-child visits, which already involve multiple administrative tasks. For a new technology intervention to be adopted in their clinic, providers agreed it would need to show clear clinical benefit while simultaneously reducing burden on clinic staff. As such, EHR integration was seen as a necessity for successful implementation of the tool across clinical settings.

Providers expressed concern about the length of time needed to complete the screening survey, both from a workflow and user perspective. Providers suspected patients and their caregivers may be unwilling to complete the entire tool, noting the form could be particularly time-consuming for individuals with low literacy levels. Providers shared that if a patient or caregiver did not have a chance to complete the survey before their visit, it may negatively affect the duration of time patients spend in exam rooms, appointment length, room turnover, and patient wait time.

Nearly all providers endorsed safety and liability concerns related to screening for suicidality. Despite the screening tool being designed to limit availability of the survey to the clinic’s hours of operation as described above, providers worried about setting up foolproof protocols for a notification system for completed screens, and assigning responsibility to staff for reviewing screens and identifying patients at risk in a timely manner to avoid positive screens being missed, or loss to follow-up:

The other thing that I think about too [is] getting a positive screen [from] someone who fills that out from home and then they don’t come to the appointment. That could be a big deal because it’s going to be our patients with mental health disorders. Generally, if a teenager has a mental health disorder, there’s a reasonable chance that a parent has a mental health disorder. If they have a mental health disorder that’s not treated, which is a lot of mental health disorders, it makes them less likely to follow through with things like appointments. So then you have a patient who’s suicidal and is out there, or who’s significantly affected and is out there, and then you can’t get ahold of them because of that stuff. That makes me scared. Now is that better than just being dumb to it? Maybe it’s better, but it makes me nervous.

Similarly, providers worried about balancing measures and the unintended harms of screening broadly for mental health without adequate time allotted during the appointment to address concerns that the screening tool may identify. A provider was concerned about the screening tool’s potential to uncover otherwise undetected mental health concerns in patients without having the resources such as behavioral health staff or referral resources to respond appropriately.

Providers raised concerns about patient privacy when using a third-party product to obtain highly sensitive protected health information. In addition, providers worried about the potential for caregivers to access a patient’s portal or survey or even to hover over their child while they complete the survey, which could in turn cause patients to answer questions inaccurately or not engage fully with the screening tool.

Accessibility was a major theme across all interviews, particularly for providers who worked with underserved populations. Providers stated it was important for the screening tool to be available in other languages, noting that if a child is fluent in English, their caregiver may be less fluent and require a version in their native language to obtain accurate collateral information. Some also wanted the screening tool to be available in other modalities, such that it would be accessible to those with poor literacy or learning disabilities. As with any new technology intervention, questions arose about health equity and inaccessibility for those without mobile devices or reliable internet access. There was general consensus that, while new technologies have potential to mitigate certain health care disparities, they also have the potential to exacerbate others by virtue of being inaccessible to many underserved populations, which is consistent with concerns in the existing literature [20].

### Structural and Organizational Considerations

The most frequently mentioned requirements for getting leadership on board with implementing a new intervention were that the intervention is efficient, enhances quality of care, increases patient satisfaction, and has proof of clinical benefit. It was also important that the intervention be simple and easy for staff to use, so as not to burden staff or negatively impact clinic workflow.

Providers identified a major barrier to implementing new technologies as lack of transparency about the processes in their institution**,** with many providers unable to identify who they would need to obtain permission from within their organization to implement a new technology within their clinic. Providers described complicated networks of clinical leadership, business executives, and representatives from information technology and their respective EHRs that all must collaborate to approve new technologies. A provider reported that they previously attempted to implement a new technology intervention in their hospital-based clinic but grew frustrated and abandoned the initiative after numerous unfruitful meetings and emails with the administration.

Some providers also mentioned public insurance documentation requirements as a barrier to using a third-party tool, as public insurance plans often require routine screenings be completed using specific forms. Providers worried some patients would have to complete the same screening questions (eg, Patient Health Questionnaire-9) twice for the same office visit—once in the enhanced screening tool, and a second time on the form required for reimbursement.

### Recommended Alterations to the Digitally Enhanced Mental Health Screening Tool

Integration of the enhanced screening tool into the EHR and automation of survey creation and distribution were thought to be the strongest facilitators necessary for implementing the tool in pediatric clinics. All 10 providers were in favor of redesigning the survey to offer all patients a subset of initial questions, and only the full screen to those patients who answered positively to those initial questions, as a means for increasing the screening tool’s efficiency.

Providers agreed that customization options such as ability to choose which screening tools to include would make the intervention more appealing to a variety of practice settings. In addition to selecting or deselecting currently available measures, providers were interested in adding screening tools for the following (in order of frequency mentioned): disordered eating, sleep, adverse childhood events, social determinants of health, attention-deficit/hyperactivity disorder (ADHD), academic distress, and demographic questions about sexual orientation and gender identity.

## Discussion

### Principal Findings

This preimplementation study aimed to elicit provider perspectives regarding proposed implementation of a digitally enhanced mental health screening tool in their respective clinical settings to inform future implementation strategies and alterations to the screening tool. While all providers stated that improving mental health screening was a high priority in their clinic and believed that the screening tool had the potential to improve such practices, many providers expressed that the screening tool may not be feasible to implement in its current form, most notably due to the tool being a third-party application without EHR integration.

As new technologies for health care information gathering, evaluation, and intervention delivery become an increasingly popular area of interest, there is a simultaneous need for improved systems interoperability to allow for exchange of information between new technology tools and existing EHRs in order for such technologies to be practical for use in real clinical settings [21]. While EHRs show provider notes and metrics from previous medical encounters, they have limited ability to pull in data and services from third-party tools that have potential to enhance quality of health care, decrease health care costs, and improve patient outcomes. The lack of connectivity between different systems and software presents a nearly impermeable barrier to implementation of new technologies, highlighting the dire need for an application-based component to health information technology to allow for adaptation of new technologies and seamless integration with existing EHRs [22]. Several large research-based hospital systems have successfully deployed questionnaires for patient reported outcomes, the results of which are directly incorporated in the electronic medical record [23]. Such initiatives can serve as a model for similar integration of screening tools such as ours and promote organizational buy-in by demonstrating that wide-scale implementation is feasible. The findings from this study further emphasize the need for enhanced systems interoperability in the health care space, and exemplify another way that improvement in this area can facilitate implementation of innovative health care technologies. Beyond the logistical barriers to implementation of the screening tool, providers identified administrative buy-in as another major barrier. Providers reported hesitancy at the administrative level due to new challenges that such technologies present, such as data privacy concerns and questions about liability in a relatively uncharted digital landscape. The findings in this study further reinforce the already identified need to address such issues at a structural or policy level [24] so that individual providers can more easily and independently trial and use new technology tools that have the potential to provide clinical benefit and improve patient outcomes without requiring system-wide changes.

### Limitations

While homogeneity within our sample allowed thematic saturation to be reached, our findings may be less generalizable to all pediatric primary care settings due to our sample being small and self-selective, comprised of providers who may be more interested in improving adolescent behavioral health services as they expressed interest in learning how a digitally enhanced screening tool could augment their existing clinical practices. While not all primary care practices will have clinician leaders interested and willing to take on the burdensome task of pioneering new interventions, early adopters are critical to intervention development and implementation such that these new tools can be optimized, scalable and feasible to disseminate more widely [25,26]. Provider feedback was also limited by the inability to use the screening tool in vivo with real patients in real time, again underscoring the importance of pilot work and a need for greater consideration regarding the process of trialing new interventions in the community (eg, flexible method designs, quasi-experimental and nonrandomized approaches that account for the need to maintain efficient workflow in practice settings).

### Conclusion

While technology interventions can help address the need for improved behavioral health support in primary care settings, numerous barriers to implementation of such tools exist. This study adds to the literature showing a need for improved health information systems interoperability for innovative technologies to be used to their greatest potential in real-world settings.

## Supplementary material

10.2196/67624 Multimedia Appendix 1 Screening Wizard provider interview guide.

## Abbreviations

ADHD: attention-deficit/hyperactivity disorder
CFIR: Consolidated Framework for Implementation Research
EHR: electronic health record
HIPAA: Health Insurance Portability and Accountability Act
PCP: primary care provider
PTSD: posttraumatic stress disorder
SW: Screening Wizard
