# Supplementary material for: Provider Perspectives on Implementing an Enhanced Digital Screening for Adolescent Depression and Suicidality: Qualitative Study
Source: JMIR Form Res. 2025 Apr 10;9:e67624. doi: 10.2196/67624 (PMC12005460; doi:10.2196/67624)
Supplement: Multimedia Appendix 1 [file formative-v9-e67624-s001.docx]

Screening Wizard Provider Interview Guide

1. Please briefly describe your professional background.
2. Please briefly describe your practice setting.
   1. How often do you see patients?
   2. What does your patient population look like?
   3. What percent would you estimate of your patients are adolescents and/or young adults?
   4. What types of problems do you see adolescents for?
   5. How many providers are in your practice?
   6. Do you have access to mental health professionals within your practice? Please describe.
   7. Do you use an electronic health record? If so, which one?
   8. Do you use a patient portal? If so, how is it used?
   9. Do patients receive screens or pre-visit questionnaires electronically?
3. Describe if and how you currently screen for depression and/or suicidality in adolescents in your practice.
   1. What is the workflow for screening (when do patients receive screening, how is it delivered, when is it reviewed, how is it documented)?
4. What kinds of high-priority initiatives or activities are already happening in your practice?
   1. How would Screening Wizard compare to these other initiatives in terms of priority?
5. What made you interested in learning more about Screening Wizard?
6. What are your initial thoughts about Screening Wizard? Is it something you would want to use in your clinic?
7. What is still unclear about to you about Screening Wizard?
8. Additional questions for providers who were able to pilot prototype of the tool with patients (if the provider did not pilot the tool, proceed to question #9):
   1. What are your thoughts after using Screening Wizard?
   2. How did Screening Wizard affect your patient care?
   3. Were there any questions that were confusing to your patients?
   4. How difficult was it to review and interpret the Screening Wizard report?
   5. Any additional thoughts?
9. Do you think you would need extra training or technical support to use Screening Wizard?
10. *“As a reminder, Screening Wizard currently screens for depression, suicide, anxiety, substance use, and mania. Among those 5 conditions, are there any that you wouldn't want to screen for? Tell me more… Are there any other conditions that you would want to screen for? Tell me more.”*
11. *“As a reminder, currently Screening Wizard exists as a unique link provided to a teen and a unique link to a parent that could be sent via text message through the Screening Wizard portal or by scanning a pre-printed QR code. We are making changes to Screening Wizard so that only adolescents screening positive for depression or suicidality would receive the rest of the questions. Currently, Screening Wizard is not embedded in the electronic health record but it is something we are hoping to achieve.”*
    1. How well does Screening Wizard fit with existing screening practices in your clinic?
    2. How does Screening Wizard compare to other similar existing programs in your setting?
12. What are some of the concerns you have about using Screening Wizard in your setting?
    1. *“Other providers have previously mentioned the following concerns: (1) logistical barriers, such as not having adequate personnel to administer the screening, (2) length of time within visit to administer the screen, (3) fear surrounding missing positive scores or not checking screening results in a timely manner, (4) not having access to mental health resources for subsequent referral and management, (5) not having enough training at the provider level to prescribe antidepressants, (6) discomfort talking with patients about suicidality, (7) the screening tool not being embedded in the electronic health record, (8) data privacy concerns and navigating the approval process to implement a new technology in their healthcare system, (9) patients not understanding [Blinded], (10) getting buy-in from other providers in their practice setting, (10) not being able to get new technologies approved by administration or other decision-makers.”*
       1. Which of these are concerns for you?
       2. Of the concerns listed, which three would be your top concerns?
       3. What would be needed to alleviate your concerns?
13. What changes or alterations do you think would be needed to facilitate implementation and utility of Screening Wizard in your clinic?
14. Who are the key influential individuals in your setting whose buy-in would be necessary to implement Screening Wizard in your practice setting?
    1. How do attitudes of different leaders vary?
    2. What kind of support would you expect from these leaders?
15. What kind of supporting evidence or proof is needed about the effectiveness of the intervention to get staff on board? Colleagues? Administrative leaders?
16. What are specific processes which could be barriers at your organization level for approving using new technologies?
17. How do you think your patients would respond to using Screening Wizard? Caregivers? Other providers in your practice? Ancillary staff? Your practice leadership?
18. Do you have any additional thoughts that you would like to share?
